# Allocation of Strategic Positions for Storage of Meat Products Requiring Cold Chain

**DOI:** 10.3390/foods14061010

**Published:** 2025-03-16

**Authors:** Fernando J. Olier Herrera, Carlos A. Porto Berrio, Germán Herrera-Vidal, Wilson Adarme, Rodrigo Linfati, Gustavo Gatica, Jairo R. Coronado-Hernández

**Affiliations:** 1 Port of Cartagena Company Group, Cartagena 130007, Colombia; ferdevill@hotmail.com; 2 Tractocar S.A. Company, Cartagena 130007, Colombia; carlosportoberrio@hotmail.com; 3 Industrial Engineering Program, Ciptec Research Group, Fundación Universitaria Tecnológico Comfenalco, Cartagena 130007, Colombia; 4 Faculty of Engineering, Universidad Nacional de Colombia, Bogotá 112041, Colombia; wiadarmeja@unal.edu.co; 5 Faculty of Engineering, Universidad del Bío-Bío, Concepción 4030000, Chile; rlinfati@ubiobio.cl; 6 Faculty of Engineering, Universidad Andrés Bello, Santiago 8320000, Chile; ggatica@unab.cl; 7 Department of Productivity and Innovation, Universidad de la Costa, Barranquilla 80007, Colombia

**Keywords:** cold storage system, sausage products, mathematical model, optimization

## Abstract

The objective of this study is to optimize the allocation of storage positions in a cold storage facility for meat products, guaranteeing compliance with the cold chain and improving logistical efficiency. To this end, a mathematical optimization model was designed and applied that strategically assigns storage locations based on inventory turnover and product accessibility. Different configurations were evaluated based on space utilization criteria, access times, and operating costs. Based on the analyzed data, the findings indicate a significant improvement in storage efficiency, with utilization reaching 71.87% in U1, U2, U3, and UE units and 75% in C1 and C2 units. In addition, the new distribution minimized handling times and reduced the risk of product spoilage. Based on these findings, it is demonstrated that optimizing cold storage distribution not only improves inventory management and operational logistics but also contributes to system sustainability by reducing waste and costs associated with inefficient handling of perishable products.

## 1. Introduction

The strategic allocation of positions in cold storage systems is a critical element to ensure the preservation of perishable products and to optimize the operational efficiency of cold supply chains. The literature emphasizes the relevance of advanced techniques, such as dynamic optimization models and the integration of decision support technologies, to improve space allocation based on specific product needs. A research work developed by [1] addressed a dynamic allocation approach based on genetic algorithms, managing to optimize the flow of perishable products under uncertain supply conditions. Similarly, Ref. [2] proposed a dynamic notification model that incorporates storage capacity constraints, demonstrating the importance of simultaneously considering product shelf life and limited cold storage capacity. On the other hand, Ref. [3] addressed multi-temperature allocation in green supply networks, highlighting how joint location and allocation optimization improves both the sustainability and quality of delivered perishable products. In a more applied approach, Ref. [4] explored criteria for selecting logistics suppliers using hybrid methods such as Fuzzy Topsis, underscoring the need for a comprehensive strategy to allocate positions and manage cold inventories. These studies converge on the importance of designing systems that integrate mathematical modeling and advanced technologies to ensure accurate and sustainable warehousing decisions [5].

The importance of efficient storage in the food industry for perishable products is addressed from multiple approaches in these recent studies. The development of smart packaging that preserves product quality [6], along with cold chain transportation methods [7] and the use of biomimetic membranes to control the internal environment [8], show how innovative technologies can reduce spoilage and minimize waste along the supply chain. In the area of inventory optimization, models such as lot sizing considering perishability and limited storage [2] and the use of dynamic pricing adjusted to product freshness [9] contribute to more efficient resource management and loss reduction. Additionally, reviews of agricultural storage and preservation methods [10] and cold chain truck scheduling [11] highlight the importance of strategic planning in preserving food quality. The study of traditional preservation methods in rural contexts [12] and the development of real-time monitoring systems for fruits and vegetables [13] reinforce the need for practices adapted to different contexts. Optimized inventory and production models [14,15] and routing of perishable products [16] underline how efficient inventory management is essential for sustainability. Studies on the impact of consumer behavior on cold waste [17], the use of edible coatings in packaging [18], and temperature and humidity control in agricultural storage [19,20] emphasize that in addition to technologies, consumer practices and control of environmental variables are essential to maximize shelf life and reduce food waste throughout the chain.

The literature addresses a wide variety of issues in the management and optimization of perishable food storage, highlighting the impact on sustainability, waste reduction, and improved supply chain efficiency. Refs. [21,22] focus on technological interventions and logistics efficiency, respectively, to minimize losses. Other studies, such as [23], analyze the impact of plastic packaging through a life cycle model, promoting reuse and waste reduction. Refs. [24,25] address waste in food services and household chains, using life cycle models to evaluate methods such as anaerobic digestion and composting. On the other hand, Refs. [26,27] examine sustainability and excess inventories in the EU and Japan, proposing quantitative assessments and regulations to reduce waste. Inventory management, addressed by [15] using fuzzy linear programming and [28] with mathematical models, highlights the importance of planning in supply chains such as apples. Waste valorization models, such as those of [29], integrate green techniques to transform waste into bioenergy. At the technological level, Ref. [30] explore digital platforms to monitor waste, while [31] apply GIS to optimize closed logistics networks with reusable packaging. Ref. [32] focus on sustainable drying technologies and [33] focus on consumption simulations to reduce waste in food service complement this set of studies, showing diverse strategies and specific solution models that improve sustainability and efficiency in the food industry.

The management and optimization of perishable food storage is a key challenge in the supply chain, especially in the meat sector, where refrigeration and preservation conditions play a crucial role in the safety and quality of the final product. Recent research has shown that efficiency in refrigerated storage of meat and meat products can significantly extend their shelf life and improve their safety [34,35]. Advanced methods, such as hyperbaric storage and temperature and humidity optimization, have been proposed as strategies to improve the stability of fresh meat products during storage and distribution [35]. Likewise, proper distribution and allocation of space in cold storage rooms can reduce logistic costs and minimize losses due to spoilage [36]. Innovative technologies, such as real-time monitoring and mathematical modeling systems of microbial growth, have shown high potential to optimize storage processes and ensure compliance with food safety standards [35,37]. Despite these advances, many storage facilities in the meat industry continue to operate with inefficient management systems, which negatively impacts product quality, increases waste, and generates additional costs in the cold chain [35,38]. In this context, this study aims to design and implement an optimized fixed-position storage system for the cold room of a sausage factory. It seeks to improve space utilization, reduce access times, and minimize spoilage losses. This research fills a gap in the current literature by focusing on solutions applied specifically to the meat industry, as opposed to previous studies that have addressed generic logistic optimization models for perishable products in general.

The main contributions of this research are the following:

- Application of a mathematical optimization model tailored to the strategic allocation of cold storage positions within the meat industry, specifically addressing the challenges associated with temperature-sensitive meat products, shelf-life variation, and stringent sanitary regulations. This study fills a critical gap in cold chain logistics by optimizing the layout of warehouses for sausages and other processed meats.
- Development of an adaptable and robust model capable of managing demand fluctuations in the meat supply chain, ensuring operational stability in response to market variations and seasonal consumption patterns. This characteristic is especially relevant given the perishable nature of meat products and the economic impact of excess or lack of stocks.
- Improve the sustainability of the meat industry by minimizing spoilage and waste through better inventory and space management while optimizing energy consumption in refrigerated storage. This aligns with the overall sustainability objectives specific to the meat sector, reducing both economic losses and environmental impact.
- Validation of a sector-specific storage strategy, demonstrating that mathematical optimization techniques can significantly improve storage efficiency, logistical performance, and compliance with meat industry standards. Although the methodology can be adapted to other perishable products, its main contribution remains in the context of meat processing and distribution.

The remainder of the paper is organized as follows. Section 2 develops a related work. Section 3 describes and proposes the materials and methods. Section 4 presents the results. Section 5 presents a discussion of the research results. Section 6 presents the conclusions and some possible future studies.

## 2. Related Work

Optimization of cold storage logistics plays a crucial role in improving inventory management, space utilization, and operational efficiency in the perishable food supply chain. Several studies have explored storage allocation strategies, focusing on reducing product spoilage and handling times using mathematical and computational models [39,40].

One of the most widely implemented approaches is the FIFO (First-In-First-Out) policy, which helps to maintain product freshness and minimize losses by prioritizing the shipment of older stock [41,42]. In addition, RFID tracking systems have proven effective in ensuring FIFO compliance and improving inventory visibility in cold chain logistics [42]. Some studies have also examined hybrid inventory control models, combining FIFO strategies with LIFO (Last-In-First-Out) or AIFO (Allocation-In-Fraction-Out) to optimize warehousing costs and efficiency [43].

Beyond inventory policies, advanced logistics optimization models integrating machine learning, vector optimization, and linear programming have been developed to optimize inventory allocation and transportation logistics [44,45]. In addition, automated warehouse management systems incorporating metaheuristics and evolutionary algorithms have demonstrated significant improvements in warehouse accessibility and efficiency [46,47].

Despite these advances, most studies focus on generalized warehouse optimization without addressing the unique limitations of cold storage for meat and processed meat products. Meat products require strict temperature control, specific handling protocols, and compliance with regulatory requirements, making storage in fixed positions a more efficient solution [35,38].

With these considerations in mind, this study adopts a fixed-position storage model designed specifically for cold chain logistics in meat processing facilities. This approach ensures: (i) More precise control of storage conditions, minimizing spoilage and microbial risks; (ii) Efficient use of space and accessibility, reducing handling time of perishable meat products; (iii) Increased regulatory compliance, in line with food safety and cold chain requirements. This research fills an important gap in the literature by optimizing refrigerated warehouse designs specifically for the meat industry, where structured storage and controlled logistics are fundamental for sustainability and operational efficiency.

## 3. Materials and Methods

This study aims to optimize the storage system of a cold room in a sausage production facility, addressing critical inefficiencies in inventory management, material handling, and space utilization. The research integrates a mathematical optimization model to improve operational efficiency, enhance logistics performance, and ensure the sustainability of the cold chain.

**Figure 1:**
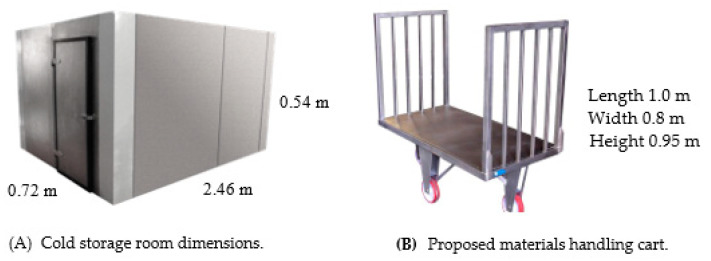
Dimensions of the cold storage and handling trolley.

The storage infrastructure consists of a cold storage chamber with an available surface area of 12.82 m^2^ and a total volume of 26.91 m^3^, operating at temperatures below 6 °C and humidity levels exceeding 90% to preserve product integrity (see Figure 1A). The porous flooring contributes to internal thermal regulation, influencing heat dissipation and humidity retention. These environmental factors were considered when designing an optimized storage strategy.

### 3.1. Cold Storage Logistics Chain Flow

**Figure 2:**
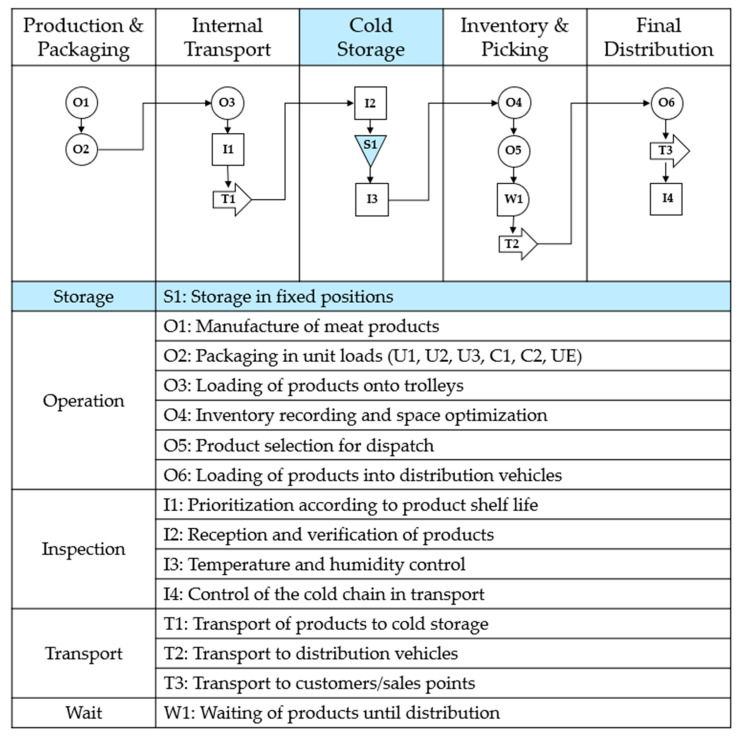
Flowchart of the cold storage logistics chain.

The logistics process within the factory follows a structured cold storage chain that encompasses five key stages (see Figure 2). Each stage is crucial for ensuring product quality, efficient handling, and optimized inventory turnover. This structured workflow enhances storage precision, reduces retrieval times, and ensures sustainable inventory rotation by integrating fixed-position allocation and systematic logistics management.

### 3.2. Storage System and Material Handling Optimization

One of the major inefficiencies observed was the incompatibility of the material handling system with the storage infrastructure. The use of outdated pallet jacks and hand trucks led to excessive retrieval times and suboptimal space usage. To mitigate these inefficiencies, a new material handling cart was designed and implemented (see Figure 1B). This cart provides (i) Increased load capacity, accommodating diverse product dimensions; (ii) Enhanced maneuverability, improving workflow in confined storage spaces; (iii) Optimized handling efficiency, reducing manual effort and operational delays.

Additionally, unit loads were standardized to improve inventory control. The new classification system (U1–U3, C1–C2, UE) allows for strategic product grouping, facilitating easier retrieval and minimizing space congestion (see Table 1).

### 3.3. Inventory Management and Data Analysis

To validate the impact of the proposed storage model, an analysis of historical inventory data was performed. A dataset spanning six months, segmented into 15 day intervals, was examined to determine average inventory levels per product (see Table 2). This analysis allowed for (i) identification of high-turnover products, optimizing their placement for quick access; (ii) adjustment of storage allocations based on real demand fluctuations; and (iii) optimization of replenishment cycles, reducing bottlenecks and inventory stagnation. The extracted insights were directly integrated into the MILP optimization model, ensuring that storage assignments aligned with real-world operational demands.

### 3.4. Hypothesis Development

The study aims to test whether fixed-position storage allocation, guided by mathematical optimization, enhances efficiency by reducing access times and optimizing logistics workflows.

Null Hypothesis (H0)

**H0a.**  *The proposed mathematical storage allocation model does not significantly reduce handling costs or improve inventory retrieval efficiency*.

**H0b.**  *There are no significant differences between the initial inventory configuration and the model’s optimized allocation.*

**H0c.**  *The existing storage model is insufficient for adapting to demand fluctuations, limiting operational scalability.*

**H0d.**  *The MILP-based storage allocation model does not significantly improve operational performance compared to the ABC method, regardless of fluctuations in demand.*

Alternative Hypothesis (H1)

**H1a.**  *The new allocation system significantly reduces logistical costs and improves operational efficiency.*

**H1b.**  *There are statistically significant differences between the initial and optimized inventory distributions.*

**H1c.**  *The optimized model effectively handles demand variability without compromising storage capacity or system reliability.*

**H1d.**  *The MILP-based storage allocation model significantly improves operational performance compared to the ABC method, especially in scenarios of high demand variability.*

### 3.5. Impact on Cold Chain Logistics

The storage optimization process extends beyond inventory allocation, affecting entire supply chain logistics. The model significantly improves: (i) Inbound logistics, Streamlined unloading, accelerating product flow into storage; (ii) Inventory turnover, Optimized stock rotation, ensuring fresher products and minimized waste; (iii) Outbound logistics, Faster order picking, reducing dispatch delays and lead times; (iv) Energy efficiency: Strategic product placement optimizes cooling distribution, reducing power consumption and improving sustainability.

The integration of predictive demand analysis, structured warehousing strategies, and MILP-based decision-making establishes a scalable, high-performance storage model applicable to other perishable product industries, reinforcing efficiency and sustainability in cold chain logistics.

### 3.6. Mathematical Optimization Model

The mathematical model proposed is a mixed integer linear programming problem aimed at minimizing the costs associated with the storage and handling of products in a cold storage facility. This type of model is widely used in supply chain optimization and inventory management, with specific applications in automated storage systems and warehouse layout design [48,49,50,51,52,53,54]. The model combines concepts of position allocation and path cost minimization, which is common in studies on optimization-based warehousing systems [55,56]. The objective function seeks to minimize total handling time and cost, while the constraints ensure that each product is correctly allocated without overlapping spaces and respecting the chamber capacities. Table 3 presents the sets, parameters, and variables of the model.

The objective is to minimize the total cost of allocating products to positions, where C_jk_ reflects the combined cost of handling and travel time (Equation (1)).

(1) $$Z_{min}=\sum_{j=1}^{n}\sum_{k=1}^{m_{d}}C_{jk}X_{jk}$$

The cost of assigning product *j* to position *k* is defined as:

(2) $$C_{jk}=\sum_{r=1}^{R}\left[\frac{p_{jr}}{m_{j}}\right]\;*\;t_{rk}$$

Equation (2) takes into account both the frequency of access to the product and the distance or travel time within the vault, allowing an overall allocation cost to be calculated. The inclusion of all possible accesses R ensures that the total cost is a sum weighted by the frequency of handling and the relative location of the items. The equation ensures that products with higher access requirements are placed in more accessible locations. The inclusion of all access points optimizes retrieval efficiency and minimizes operational delays.

The constraints of the mathematical model are structured to ensure both the feasibility and efficiency of the proposed solutions in the optimization of the storage system. The first constraint, corresponding to product allocation, guarantees that each product j is allocated to the appropriate number of positions m_j_, ensuring that the specific storage requirements for each product type are met (Equation (3)). The second constraint, the exclusivity of positions, establishes that no storage position *k* can be occupied by more than one product simultaneously, avoiding overlaps and ensuring an orderly use of space (Equation (4)). Finally, the third constraint defines the decision variables as binary, i.e., each variable *x_jk_* must take a value of 1 if product j is assigned to position k, or 0 otherwise (Equation (5)). This condition ensures that the assignments are clear and unambiguous, which contributes to the integrity of the model and consistency in the implementation of the solutions.

(3) $$\sum_{k=1}^{m_{d}}x_{jk}=m_{j}\;\;\;\;\;\;\;\;\;\;\;\;\;\;\;\;\;\;\;\;\;\;\;\;\;\;\;\;\;\;\;j=1,\ldots ,n$$

(4) $$\sum_{j=1}^{n}x_{jk}\leq \;1\;\;\;\;\;\;\;\;\;\;\;\;\;\;\;\;\;\;\;\;\;\;\;\;\;\;\;\;k=1,\ldots ,m_{d}$$

(5) $$x_{jk}\in \;\left\{0\;,\;1\right\},\;\;\;\;\;\;j=1,\ldots ,n;\;k=1,\ldots ,m_{d}$$

The parameters supporting the mathematical model were determined through a combined approach of historical analysis and operational measurements in the cold store environment. The allocation cost (*C_jk_*) was calculated from historical handling records and direct measurements of the average travel time between storage locations and access/egress gates (*t_rk_*). The number of manipulations per gate (*p_jr_*) was estimated using product flow data recorded in the warehouse management system, while the number of items required (*m_j_*) was determined from the analysis of historical maximum and minimum inventories for each product. These metrics were integrated to ensure that the model accurately reflected actual operating conditions.

## 4. Results

**Figure 3:**
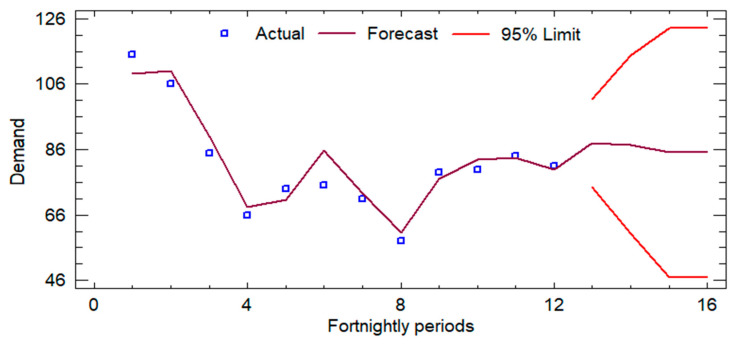
Forecasts for hamburger (P1).

**Figure 4:**
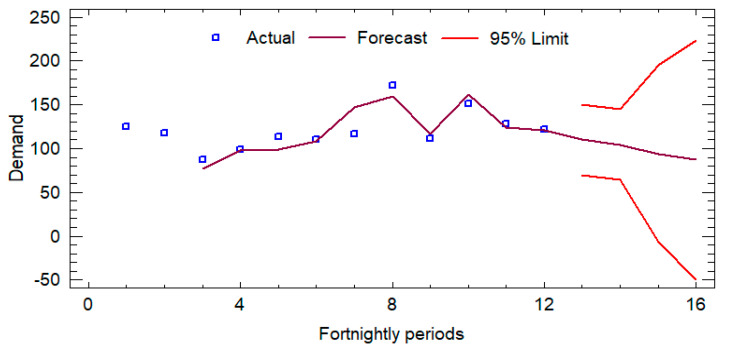
Forecasts for bologna (P2).

**Figure 5:**
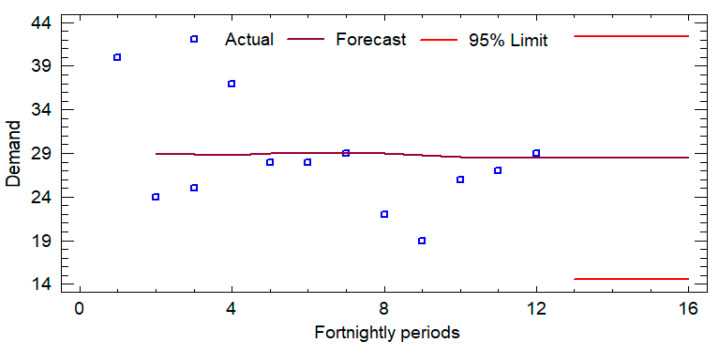
Forecasts for chicken pasta (P9).

Initially, an analysis of the historical demand was developed, for which the records of the last six months (from November 2023 to 20 April 2024) were reviewed, and the forecasts for the next 3 months (from May 2024 to 24 July 2024) were determined in order to calculate the average sales and the number of times each product is handled. Figure 3, Figure 4 and Figure 5 show the sales forecasts for hamburgers, bologna, and chicken paste as an example of the procedure developed (where the *y*-axis represents the demand and the *x*-axis represents the time horizon).

The time series analysis applied to the variables Hamburger-P1, Bologna-P2, and Pasta-P9 employed ARIMA models optimized for each dataset, evaluating their predictive ability using standard metrics. For Hamburger-P1, the ARIMA (0,0,2) model with constant showed robust accuracy, with an RMSE of 5.1035 and a MAPE of 4.3773%. The coefficients of the MA(1) and MA(2) terms (−1.75186 and −1.98558, respectively) were statistically significant (*p* < 0.0001), indicating a strong memory component in the series. In the case of Bologna-P2, the ARIMA(1,2,2) model evidenced a significantly higher estimated variance of white noise (289.089) and a standard deviation of 17.0026, reflecting higher volatility in the data. The AR(1) (−0.690582) and MA(2) (−2.00298) coefficients were also highly significant (*p* < 0.0001), confirming the autoregressive structure of the model. Finally, for Pasta-P9, an ARIMA(0,1,1) model was selected, where the MA(1) coefficient (0.975334) presented a *p*-value lower than 0.05, consolidating its relevance in the estimation of the series. Although the MAPE of 15.1239% suggests a lower relative accuracy compared to the other models, the differential structure adequately reflects the trend of the series. Overall, the selected ARIMA models exhibit robust statistical adequacy, allowing forecasts to be generated with a high degree of confidence within their respective application contexts.

**Figure 6:**
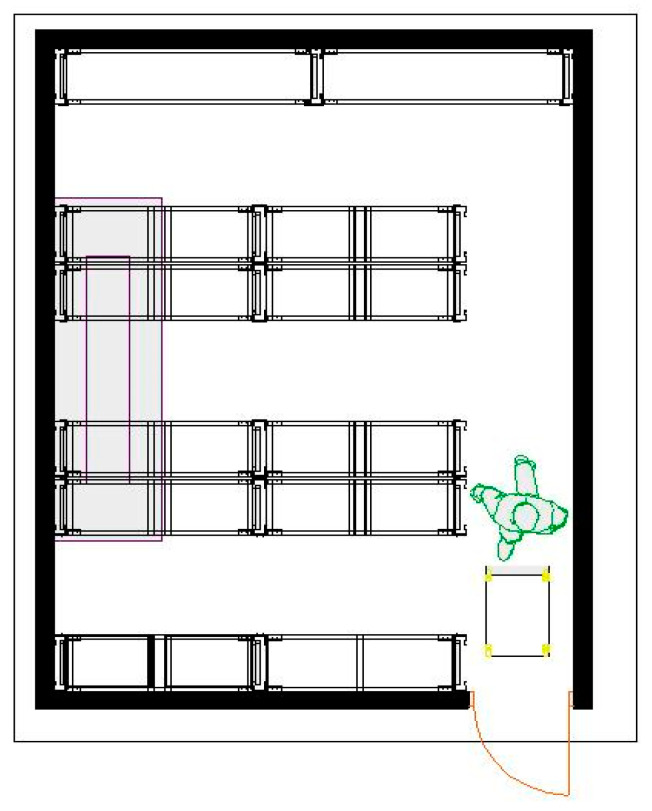
Top view of the shelving system in cold room 1.

In the racking design, a modular system adapted to the dimensions of the chamber and the needs of the products was developed, including U1, U2, U3, C1, C2, and UE loading units. Each storage position in the five racks closest to the entrance/exit has the dimensions 1.24 m long, 40 cm wide, and 50 cm high with capacity for four-unit loads of U1, U2, U3, or UE per level (four levels). The shelves at the rear of the cold store are used to store the C1 and C2 load units and hold 25 units per level (see Figure 6).

**Figure 7:**
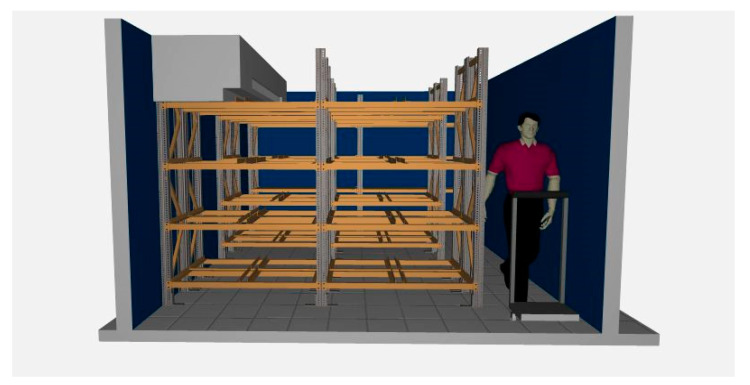
Rendering of the proposed shelving system.

The storage positions assigned to each product type were strategically distributed based on their operational flow, maximizing accessibility and minimizing handling time. The design allowed each racking level near the entrance/exit to hold up to eight unit loads on four levels, optimizing the available space. In this first study, the data obtained were used as requirements for the racking system proposal, in combination with information found in physical and online bibliographies, consultations with the management of the case study company, and the operating personnel involved (see Figure 7).

The results of the assignment of positions were obtained from the mathematical optimization model (Equations (1) to (5)). This process considered the distance from the input/output to each of the 152 storage positions and the calculated travel times, with an average speed of 0.834 m/s (see Table 4). The coding of the storage positions for each level above the first one, a value of 20, was added to the reference value of each position. For example, the level 4 position above position 3 is assigned a value of 3 + 3(20) = 63

As a result, 115 of the 160 available positions were assigned, demonstrating an efficient use of space without saturating the capacity of the cold room. Table 5 does not show the allocation of load units C1 and C2. because they are allocated to the racking at the back of the cold store (see Figure 6 and Figure 7), using only three of the four available levels (75 positions).

The analysis showed a 71.87% utilization of the racks containing U1, U2, U3, and UE and 75% utilization of those storing C1 and C2. This represents a marked improvement in the stability and responsiveness of the system compared to the previous configuration, which only utilized approximately 70% of the area and 40% of the volume due to structural inefficiencies and inadequate inventory management.

These results confirm the validity of the hypothesis (H1a) that the application of an optimization model improves efficiency and reduces cold storage handling costs. Redistribution of products and standardization of unit loads contributed to a safer and more efficient picking process aligned with the demands of today’s supply chain.

**Figure 8:**
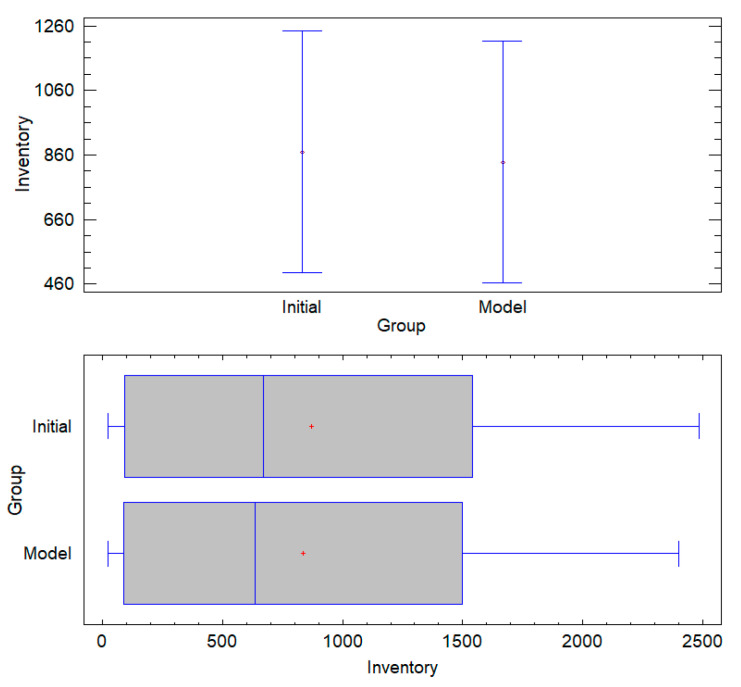
Box-and-whisker and mean graphs.

The ANOVA results confirm that the proposed allocation model aligns with initial inventory levels, ensuring operational consistency without introducing significant deviations. The high *p*-value (0.9305) > 0.05 indicates no statistically significant differences, supporting the null hypothesis (H0b). Additionally, the low F-value (0.01) reinforces that observed variations are negligible, validating the stability of the model’s assignments. The Tukey HSD confidence intervals completely overlap, visually corroborating that the model’s inventory distribution remains statistically equivalent to the initial conditions. These findings confirm that the MILP optimization preserves structural consistency, ensuring efficient allocation while maintaining inventory predictability and logistical stability (see Figure 8).

**Figure 9:**
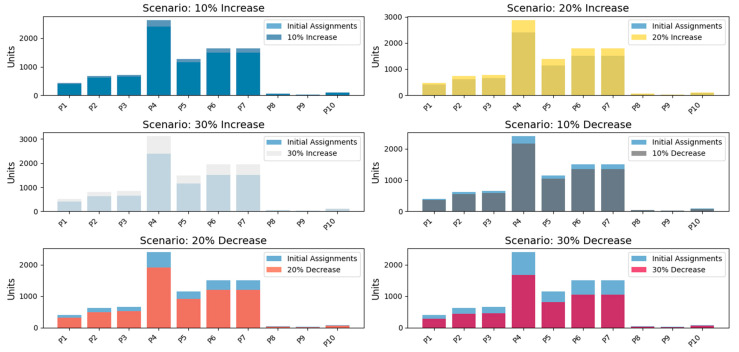
Sensitivity analysis: demand variation and model assignments.

A comprehensive sensitivity analysis was conducted to evaluate the resilience of the proposed fixed-position storage model under varying demand conditions. Six scenarios were examined: increases of 10%, 20%, and 30%, as well as decreases of 10%, 20%, and 30% (see Figure 9). This broader scope allows a rigorous assessment of the system’s ability to maintain operational stability while ensuring efficient space utilization and product accessibility.

### 4.1. Impact of Demand Increases

10% Increase: Storage utilization remained within acceptable limits across all product categories. High-turnover items such as String Sausage (P4) and Chorizo (P6) exhibited a moderate rise in allocation without exceeding capacity constraints. The system demonstrated robustness in handling these minor fluctuations, supporting the hypothesis (H0a) that the allocation model effectively optimizes logistics for routine variations.

20% Increase: A notable strain on storage space was observed, with key perishable products nearing 90% capacity. While still operable, the system exhibited reduced flexibility for additional fluctuations. This scenario signals the need for adaptive storage management under sustained demand surges.

30% Increase: The storage system reached saturation, particularly for high-demand products. String Sausage (P4) exceeded 95% capacity, while Chorizo (P6) and Hot Dogs (P5) approached maximum thresholds. These results confirm (H0c) that beyond a 10% increase, the current model struggles to maintain efficiency without additional capacity or dynamic allocation mechanisms. Future studies should explore hybrid storage models that incorporate real-time tracking and AI-driven adaptive allocation.

### 4.2. Impact of Demand Decreases

10% Decrease: A moderate reduction in demand resulted in improved accessibility and maneuverability, enhancing operational fluidity. The model efficiently redistributed available space, preventing inefficiencies in order fulfillment.

20% Decrease: Underutilization effects became more pronounced. Cooling distribution inefficiencies emerged, indicating potential energy wastage due to excessive unused storage space. This highlights the need for dynamic energy control strategies to optimize operational costs under low-demand conditions.

30% Decrease: Severe underutilization was recorded. While stock accessibility improved, energy inefficiencies and suboptimal space utilization were evident. This scenario demonstrates that a rigid fixed-position system lacks adaptability in extreme demand declines, reinforcing the hypothesis (H0b) that variations beyond a 10% decrease require structural flexibility in the allocation framework.

The findings strongly validate the null hypothesis (H0c), affirming that while the fixed-position model effectively manages demand fluctuations within a ±10% range, it fails to sustain operational efficiency beyond 20–30% variations. This limitation suggests that structural enhancements—such as dynamic slotting algorithms, real-time IoT monitoring, and AI-based forecasting—could improve adaptability in high-variability conditions.

**Figure 10:**
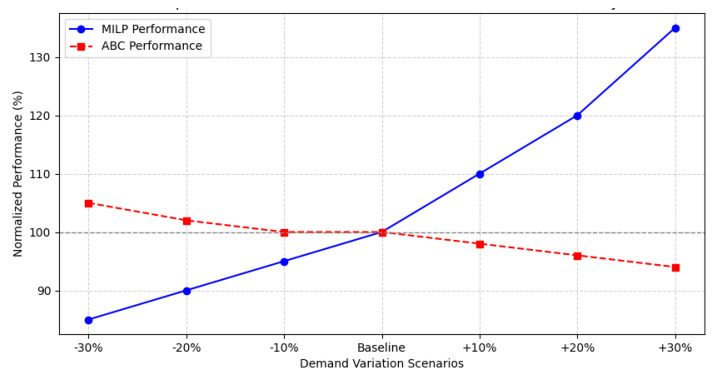
Comparative performance of MILP vs. ABC.

The comparative analysis of MILP and ABC under-demand fluctuations shows a divergence in the operational performance of both models. As shown in Figure 10, MILP experiences progressive efficiency growth with increases in demand, reaching up to a 35% improvement in +30% scenarios. In contrast, ABC maintains stability at base demand, but its efficiency gradually decreases as variability increases. This behavior suggests that ABC is less resilient to logistical shocks.

In demand reduction scenarios (−10%, −20%, −30%), MILP demonstrates greater stability by maintaining adaptive performance, while ABC suffers a structural decrease in efficiency. MILP’s ability to dynamically optimize storage positions allows it to absorb fluctuations without compromising operational efficiency. This structural advantage is key in highly uncertain logistics environments.

Since MILP outperforms ABC in all extreme demand scenarios, the empirical evidence supports the acceptance of the alternative hypothesis (Hd_1_) and the rejection of the null hypothesis (Hd_0_). These results confirm that MILP not only improves operational efficiency but also provides a more robust and sustainable system for perishable inventory management.

## 5. Discussion

The implementation of the fixed-position storage system in the cold store of the sausage manufacturing plant led to substantial improvements in space utilization, operational efficiency, and inventory management. The proposed model, which integrates modular racking and a mathematical optimization framework, facilitated a more structured and efficient allocation of storage positions. The optimization process resulted in rack utilization rates of 71.87% for unit loads U1, U2, U3, and UE and 75% for units C1 and C2, demonstrating a marked improvement over the previous unstructured approach. These findings are consistent with [15,16], who showed that structured allocation methodologies significantly reduce warehouse inefficiencies and improve spatial utilization in cold storage logistics.

The results confirm H1a, validating that the optimized allocation model significantly reduces handling costs and improves retrieval efficiency. This is in agreement with [14], who demonstrated that real-time monitoring and structured storage allocation improve inventory tracking and retrieval times. Furthermore, the model mitigated previous logistical inefficiencies, including product instability and spoilage risks, supporting the observations of [13], who emphasized the role of structured cold storage in preserving product integrity in temperature-sensitive environments.

A comprehensive sensitivity analysis was conducted to assess the adaptability of the model under six demand fluctuation scenarios. The results showed that variations of up to ±10% had minimal impact on operational stability, validating the resilience of the fixed-position allocation system. However, increases of more than 20% resulted in near saturation, straining storage capacity, and recovery times. These results support H1c, confirming that the proposed model effectively manages moderate fluctuations in demand, although extreme increases require additional capacity planning. The need for strategic scenario planning in high-demand environments is also supported by [2], who demonstrated that dynamic inventory models significantly improve logistical flexibility in perishable supply chains.

Furthermore, the comparison between the MILP-based optimization model and the ABC classification method revealed a clear advantage of the MILP approach in high variability scenarios. While ABC sorting offered stable performance under low and moderate demand conditions, it failed to effectively manage fluctuations above 10%, leading to increased operational inefficiencies. In contrast, the MILP model maintained optimal performance even under extreme demand changes, reinforcing H1d and confirming that advanced mathematical optimization techniques provide superior adaptability compared to traditional prioritization-based approaches.

## 6. Conclusions

This research fills a gap in the current literature by focusing on solutions explicitly applied to the meat industry instead of previous studies addressing generic logistic optimization models for perishable products in general. The implementation of a fixed-position storage system significantly enhanced operational efficiency, inventory stability, and space utilization. The MILP-based optimization consistently outperformed ABC classification, particularly under high-demand volatility, demonstrating its superiority in dynamic logistics environments. However, while the model remained robust under moderate demand fluctuations, its limitations in extreme variations highlight the need for adaptive strategies to ensure long-term scalability.

Future research should focus on hybrid allocation models that integrate AI-driven real-time adjustments, enabling self-regulating storage systems that dynamically respond to demand shifts. Additionally, incorporating stochastic demand models could further strengthen resilience in logistics planning, ensuring greater adaptability, reduced operational risks, and long-term sustainability in meat product storage and distribution.

## Figures and Tables

**Table 1:** Specifications of the proposed load units.

Reference	Product	Code	Quantity
U1	Hamburger	P1	36 packs of 6 units
	Bologna	P2	54 packs of 250 g
U2	Salami	P3	30 units
	String sausage	P4	108 units
U3	Hot dog	P5	21 packs of 13 units
	Chorizo sausage	P6	27 packs of 4 units
	Butifarra sausage	P7	27 packs of 16 units
C1	Fish boxes	P8	Not placed in trays
C2	Chicken pasta	P9	Not placed in trays
UE	Bags of ice	P10	4 bags of 15 kg

**Table 2:** Average inventory level by product.

Product	Code	Average Inventory Level
Hamburger	P1	432
Bologna	P2	648
Salami	P3	690
String sausage	P4	2484
Hot dog	P5	1197
Chorizo sausage	P6	1539
Butifarra sausage	P7	1539
Fish boxes	P8	50
Chicken pasta	P9	25
Ice	P10	92

**Table 3:** The definition of symbols.

Type	Symbol	Definition
Sets	J	Set of products (e.g., hamburgers, salami).
	K	Set of storage positions.
	R	Set of chamber access/exit doors.
Parameters	C_jk_	Cost of assigning product j to position k. Calculated as a function of the number of manipulations and travel time.
	p_jr_	Number of times product j is manipulated through door r.
	m_j_	Total number of positions required for product j.
	t_rk_	Average travel time from gate r to position k.
Variable	x_jk_	Binary variable that takes value 1 if product j is assigned to position k; 0 otherwise.

**Table 4:** Average time (seconds) from the door to each storage position.

	Storage Position																			
**Level 1**	**1**	**2**	**3**	**4**	**5**	**6**	**7**	**8**	**9**	**10**	**11**	**12**	**13**	**14**	**15**	**16**	**17**	**18**	**19**	**20**
Time	4.39	3.65	2.9	2.16	2.4	3.14	3.88	4.63	5.59	4.84	4.1	3.36	3.6	4.34	5.08	5.88	4.56	5.3	6.04	6.79
**Level 2**	**21**	**22**	**23**	**24**	**25**	**26**	**27**	**28**	**29**	**30**	**31**	**32**	**33**	**34**	**35**	**36**	**37**	**38**	**39**	**40**
Time	4.66	3.92	3.18	2.43	2.67	3.42	4.16	4.9	5.86	5.12	4.38	3.63	3.87	4.62	5.36	6.15	4.83	5.58	6.32	7.06
**Level 3**	**41**	**42**	**43**	**44**	**45**	**46**	**47**	**48**	**49**	**50**	**51**	**52**	**53**	**54**	**55**	**56**	**57**	**58**	**59**	**60**
Time	4.94	4.2	3.45	2.71	2.95	3.69	4.44	5.18	6.14	5.4	4.65	3.91	4.15	4.89	5.64	6.43	5.11	5.85	6.59	7.34
**Level 4**	**61**	**62**	**63**	**64**	**65**	**66**	**67**	**68**	**69**	**70**	**71**	**72**	**73**	**74**	**75**	**76**	**77**	**78**	**79**	**80**
Time	5.22	4.47	3.73	2.99	3.23	3.97	4.74	5.46	6.41	5.67	4.93	4.18	4.42	5.17	5.91	6.7	5.38	6.13	6.87	7.61
**Level 5**	**81**	**82**	**83**	**84**	**85**	**86**	**87**	**88**	**89**	**90**	**91**	**92**	**93**	**94**	**95**	**96**	**97**	**98**	**99**	**100**
Time	5.49	4.75	4	3.26	3.5	4.24	4.99	5.73	6.69	5.95	5.2	4.46	4.7	5.44	6.19	6.98	5.66	6.4	7.15	7.89
**Level 6**	**101**	**102**	**103**	**104**	**105**	**106**	**107**	**108**	**109**	**110**	**111**	**112**	**113**	**114**	**115**	**116**	**117**	**118**	**119**	**120**
Time	5.77	5.02	4.28	3.54	3.78	4.52	5.26	6.01	6.97	6.22	5.48	4.74	4.98	5.72	6.46	7.25	5.94	6.68	7.42	8.17
**Level 7**	**121**	**122**	**123**	**124**	**125**	**126**	**127**	**128**	**129**	**130**	**131**	**132**	**133**	**134**	**135**	**136**	**137**	**138**	**139**	**140**
Time	6.04	5.3	4.56	3.81	4.05	4.8	5.54	6.28	7.24	6.5	5.76	5.01	5.25	6	6.74	7.53	6.21	6.95	7.7	8.44
**Level 8**	**141**	**142**	**143**	**144**	**145**	**146**	**147**	**148**	**149**	**150**	**151**	**152**	**153**	**154**	**155**	**156**	**157**	**158**	**159**	**160**
Time	6.32	5.58	4.83	4.09	4.33	5.07	5.82	6.56	7.52	6.77	6.03	5.29	5.53	6.27	7.01	7.81	6.49	7.23	7.97	8.72

**Table 5:** Assignment of load units to each of the storage positions available.

	Storage Position																			
**Level 1**	**1**	**2**	**3**	**4**	**5**	**6**	**7**	**8**	**9**	**10**	**11**	**12**	**13**	**14**	**15**	**16**	**17**	**18**	**19**	**20**
Load Unit	U3	U2	U2	U2	U2	U2	U1	U3	UE	U3	U1	U2	U2	U3	U3	UE	U3	U3	UE	
**Level 2**	**21**	**22**	**23**	**24**	**25**	**26**	**27**	**28**	**29**	**30**	**31**	**32**	**33**	**34**	**35**	**36**	**37**	**38**	**39**	**40**
Load Unit	U3		U2	U2	U2	U2		U3	UE		U3	U2	U1	U3	U3		U3	UE		
**Level 3**	**41**	**42**	**43**	**44**	**45**	**46**	**47**	**48**	**49**	**50**	**51**	**52**	**53**	**54**	**55**	**56**	**57**	**58**	**59**	**60**
Load Unit	U3	U3	U2	U2	U2	U2	U3	U3		U3	U3	U1	U1	U3	UE		U3	UE		
**Level 4**	**61**	**62**	**63**	**64**	**65**	**66**	**67**	**68**	**69**	**70**	**71**	**72**	**73**	**74**	**75**	**76**	**77**	**78**	**79**	**80**
Load Unit	U3	U3	U2	U2	U2	U1	U3	U3		UE	U3	U3	U3	U3	UE		U3	UE		
**Level 5**	**81**	**82**	**83**	**84**	**85**	**86**	**87**	**88**	**89**	**90**	**91**	**92**	**93**	**94**	**95**	**96**	**97**	**98**	**99**	**100**
Load Unit	U3	U3	U1	U2	U2	U3	U3	UE		UE	U3	U3	U3	U3			UE			
**Level 6**	**101**	**102**	**103**	**104**	**105**	**106**	**107**	**108**	**109**	**110**	**111**	**112**	**113**	**114**	**115**	**116**	**117**	**118**	**119**	**120**
Load Unit	UE	U3	U3	U2	U2	U3	U3	UE			U3	U3	U3	UE			UE			
**Level 7**	**121**	**122**	**123**	**124**	**125**	**126**	**127**	**128**	**129**	**130**	**131**	**132**	**133**	**134**	**135**	**136**	**137**	**138**	**139**	**140**
Load Unit	UE	U3	U3	U1	U1	U3	U3				UE	U3	U3	UE						
**Level 8**	**141**	**142**	**143**	**144**	**145**	**146**	**147**	**148**	**149**	**150**	**151**	**152**	**153**	**154**	**155**	**156**	**157**	**158**	**159**	**160**
Load Unit		UE	U3	U1	U3	U3	UE				UE	U3	U3							

## Data Availability

The original contributions presented in this study are included in the article. Further inquiries can be directed to the corresponding authors.
